# On Infantile Paralysis

**Published:** 1863-11

**Authors:** Holmes Coote

**Affiliations:** Senior Assistant-Surgeon to St. Bartholomew’s Hospital, and Assistant-Surgeon to the Royal Orthopædic Hospital, Lecturer on Surgery to St. Bartholomew’s Hospital


### ON INFANTILE PARALYSIS.

By HOLMES COOTE, Esq., F.R.C.S., Senior Assistant-Surgeon to St. Bartholomew's Hospital, and Assistant-Surgeon to the Royal Orthopædic Hospital, Lecturer on Surgery to St. Bartholomew's Hospital.

Reprinted from the "London Lancet."

The description of the disease termed "infantile paralysis" is, I think, generally defective in two particulars; first, it is regarded as a morbid affection standing somewhat apart from other diseases of the nervous system; secondly, the description does not include those final and secondary changes in the muscular and osseous systems which produce contractions of limbs, deformities, or even complete loss of power.

The Orthopædic Hospital affords a very ample field for the investigation of this subject, about 80 of every 1000 patients being instances of the affection in some one of its varied forms; and I must here state, that the cases which come under treatment appear to me to be the survivors of a yet larger number, many of whom have perished from the violence of the symptoms at the first attack. I cannot, therefore, look upon it as a "disease not dangerous to life;" although it is true that many survive, bearing upon them, nevertheless, in their withered and contracted limbs the traces of the severity of the shock they have sustained.

In speaking of this class of nervous affections, I must limit the terms of "infantile paralysis" to one of the effects produced by the functional disturbance of the nervous centres; for, as must hereafter be shown, muscular atrophy and general loss of temperature and power express, in a very rough way, the complications with which we have to deal. Atrophy may be limited to one muscle, or to a set of muscles; there may be contraction of the limb either with or without atrophy; the direction of the contraction varies extremely.

Respecting the condition of the brain and spinal cord, I need scarcely observe that the absence of any recognized morbid change of structure in those cases in which we have the opportunity of making a *post mortem* examination is rather to be regarded as an instance of our defective means of observation than as a proof that no such morbid change exists. Of the truth of this statement, tetanus offers a well-known proof. Morbid anatomy has hitherto revealed nothing satisfactory to explain the phenomena which occur during life. The same remark applies in general to cases of infantile paralysis. But in one case a deposit of tuberculous matter was found in the membranes of the brain about the cerebellum; and in another, an impacted calculus seemed the cause of the disturbance by its reflex irritation. The most common exciting cause is the irritation of first dentition; but I have known the same effect to be produced by the second dentition, and have witnessed symptoms of analagous character, though not so strongly marked, in the adult. A young lady, twenty-two years of age, whose teeth were crowded together and partly decayed, experienced occasional attacks of numbness and loss of power in one upper extremity, recurring at intervals, until she had been relieved by an experienced dentist of the stumps of a number of decayed teeth, which had been the source of pain. But the attack may be quite sudden, without any recognized premonitory symptom. Thus an infant at the breast will appear to have momentary faintness, or may be taken up by the nurse as usual after an apparently uninterrupted night's rest, and in both instances the limbs may be found paralyzed.

On May 7th, 1863, I saw a boy, aged 8, at the Orthopædic Hospital, in whom the left lower extremity was smaller in circumference, shorter, and colder that the opposite, the foot being in the position known as equino-varus. His mother gave the following history:—At two years of age, when to all appearance well, he suddenly fell down stairs from a landing whereon he was playing. When taken up, it was found that the limb had lost all power, and he has never walked since.

On the same day I saw another boy, Henry M——, aged 4, suffering from talipes equino-valgus of the left limb. The mother said that the child began to walk when about twelve months old. He was then, to use her own words, "taken off his feet" by vomiting and purging. For two years he was quite unable to walk. He was galvanized at another hospital, but without avail. After a time, he became stronger in the back, and could sit up; finally, he recovered the use of all the limbs, except the left lower extremity, which has continued weak, cold, and deformed. The limb is smaller than the opposite in circumference by a quarter of an inch, and shorter by half an inch.

The same effects have been referred to the influence of eruptive disorders on the frame. On May 18th, 1863, I saw a boy, named Richard G——, aged 6, suffering from talipes equinus. The mother said that six weeks after having had the measles he went to bed as usual. The next morning she found the right leg completely paralyzed. After a time, some power returned in the muscles of the calf, and the heel was drawn upwards. The limb was smaller in circumference, but not materially shorter.

I do not believe that "talipes equinus" is ever congenital as a deformity. Now, there are two classes of cases, which, though allied as indicating disturbance of the nervous centres, and in both instances followed by contractions of the limbs, are yet separated by important points of difference. In the first, the limbs are spasmodically contracted, the thighs and legs bent, the heel raised, and the movements are irregular, but there is neither atrophy of the limb nor diminution of temperature. In the second, the heel may be raised or the foot otherwise turned; but the limb is shorter, smaller, colder, and wasted. In the first, the muscular degeneration and conversion into fat is a slow process, extending over many years. In the second it is rapid, the growth and development of the limb being arrested from the very moment of the stroke. I believe the first to be an instance of disturbance of the control of the will over muscular power from *cerebral* irritation, such as would be excited by the deposit of tuberculous matter in the membranes about the cerebellum; the second to be an instance of lesion of the *spinal cord.* And, although no very clear line of demarcation can in all cases be drawn between the two, yet we find that in the first class of cases the patients are often morbidly excitable, irritable, and prone to laugh or cry; while in the second class, after the first symptoms have subsided, the functions of the brain do not seem to be affected.

I fancy that in these cases of cerebral disturbance there is often a congenital defect, the development of the brain and the manifestation of the intellect being imperfect. I am now attending the daughter of a lady, whose case illustrates this point. The child is about 10 years of age, and she was put under my care in consequence of her ungainly walk, the knees being slightly bent, and the feet having an inclination inwards. I found that the muscles of the calf were so much contracted that at the foot was held at right angles to the leg, and the heels came with difficulty to the ground. The control of the will over the muscles generally was imperfect. The child could learn a short lesson, but was not studious; was subject to violent fits of temper, and never had shown affection for any about her. The general aspect was such as warranted a prediction that these peculiarities might become stronger with age.

The ultimate effects of infantile paralysis, dependent on lesion of the spinal cord, as it affects the limbs, are as follows:—

In by far the greater number of cases the loss of power is in the lower limbs. At the commencement, the paralysis may be general and complete, but usually all the limbs save one recover as mysteriously as they were attacked. Neither sex nor side of the body seems to exercise any influence. Boys and girls, right and left side of the body, suffer equally. But the paralysis is rarely complete: the foot rarely hangs powerless; usually the muscles of the calf retain some power, and pull up the heel (talipes equinus paralyticus); or the foot may incline inwards (talipes equino-varus); or the muscles of the calf and the peronei may pull it outwards (talipes equino-valgus); or, finally, the anterior tibial muscles may overcome the paralyzed muscles of the calf, producing talipes calcaneus.

As patients grow up, the great annoyance which they experience is the liability to sprains. All the ligaments are weak and elongated; and if a person so afflicted tread on a stone or any rough body, he gives the limb a twist which lays him up for days.

The affection may be limited to an upper extremity, in which case, usually, the deltoid muscle becomes paralyzed, and the humerus drops from the socket; or, in rarer instances, the muscles of the upper arm become wasted, the forearm being well formed as usual.

There are cases in which the affection comes on slowly and progressively; others in which first one limb and then another is attacked, but these are comparatively rare. I saw a boy, aged sixteen months, in whom the right heel had been drawn up when the child was five months old. That attack passed away; at the present time (May 18th, 1863) the left heel is contracted.

In all these cases, the treatment consists in an attempt to remove the source of irritation, whether it be seated in the brain or spinal cord, or whether there be any amount of reflex irritation. As a general rule, tonics are not indicated; but purgatives and small doses of tartar emetic often relieve symptoms. The temperature of the limb must in all cases be carefully maintained; and when, finally, the contraction has become permanent, the proper tendons should be divided, and elongated by subcutaneous tenotomy and extension, the foot being held in proper position by the aid of irons. The details of this treatment would lead me into particulars which are already well known to the profession, and which require modification according to the nature of the case.

Queen Anne Street, Cavendish Square, 1863.

